# Glaucoma Progression Diagnosis: The Agreement between Clinical Judgment and Statistical Software

**DOI:** 10.3390/jcm11195508

**Published:** 2022-09-20

**Authors:** Gloria Roberti, Manuele Michelessi, Lucia Tanga, Luca Belfonte, Laura Maria Del Grande, Marisa Bruno, Francesco Oddone

**Affiliations:** IRCCS—Fondazione Bietti, Via Livenza 3, 00198 Rome, Italy

**Keywords:** glaucoma, visual field, glaucomatous field defects

## Abstract

Background: To explore the agreement between clinical judgment and Guided Progression Analysis II (GPAII) in the evaluation of visual fields (VF) progression in patients with glaucoma. Methods: Three glaucoma experts and three general ophthalmologists were asked to rate the VF series by classifying them as progressive through the observation of the overview report. The agreement between clinical judgment and GPAII event analysis (EA) and trend analysis (TA) was assessed by Cohen statistic. The sensitivity and specificity of clinical judgment in detecting the presence of progression was evaluated considering the results of GPAII as the reference standard. Results: 66 VF series were included in the study. Glaucoma experts, general ophthalmologists, GPAII EA, and GPAII TA found progression in 39%, 38%, 15%, and 21% of the VF series (*p* < 0.05). The clinical judgment of glaucoma experts and general ophthalmologists was discordant with GPAII EA in 27.2% and 28.7% (k = 0.35, 95% CI 0.15–0.56 and k = 0.30, 95% CI 0.09–0.52) and with GPAII TA in 21.2% and 25.7% of the VF series examined (k = 0.51, 95% CI 0.31–0.72 and k = 0.41, 95% CI 0.18–0.62). Considering the GPAII EA and TA as reference standard, glaucoma experts showed a sensitivity of 90% and 92.8% and a specificity of 69.6% and 75%, while general ophthalmologists showed a sensitivity of 80% and 78.5% and a specificity of 69.6% and 73%. Conclusions: The agreement between clinical judgment and GPAII ranges from fair to moderate. Glaucoma experts showed better ability than general ophthalmologists in detecting VF progression.

## 1. Introduction

Glaucoma is a chronic progressive disease of the optic nerve characterized by retinal ganglion cell loss and relative visual field (VF) defects [1]. The treatment goal for this disease is to slow down the progression of the functional damage which would lead to blindness [2]. Therefore, in clinical practice, identifying signs of progression is pivotal to follow up patients and to manage their condition.

Visual field progression is mostly evaluated by clinical judgement of the overview report and by statistical software available in the perimeter [3].

The clinical judgment involves the manually review of serial visual field tests, looking at the available plots (grey scale, threshold absolute values, total and pattern deviation plots), the VF indices (mean deviation, MD, pattern standard deviation, PSD, visual field index, VFI), the severity of the sensitivity loss, the general or local depression, the topography of the defect, and its worsening in deepness or extension. Non-standardized criteria are applied to assess progression.

The most commonly used software is the Guided Progression Analysis II (GPA) in the Humphrey Field Analyzer (Carl Zeiss Meditec, Dublin, CA, USA).

The GPAII provides both an event analysis (EA) based on the Early Manifest Glaucoma Trial (EMGT) protocol and a trend analysis (TA) [4].

For the EA, the GPAII compares all the points tested in the VF with respect to two baseline exams and indicates a “possible” progression when the same three points (even if not contiguous) show a worsening in two consecutive exams, while indicating a “likely” progression if the same three points show a worsening in three consecutive exams.

For the TA, the GPAII performs a linear regression analysis over time of the VFI, which expresses visual function as a percentage of a perimetrically normal age-corrected VF, of at least five exams over three years and indicates whether the slope of the regression line is significant or not [5].

The GPAII is less time consuming compared to clinical judgment because it provides an immediate result about the presence of visual field progression or not. Furthermore, it considers a change significant if it is greater than the test-retest expected variability, so the results are not affected by the “within test” variability (short-term fluctuation) or the “between test” variability (long term fluctuation), both of which are known to be increased above normal in glaucoma and are difficult to detect for clinicians by looking at the overview report [6,7].

The purpose of this study is to evaluate the agreement in recognizing the signs of VF progression between the clinical judgment of glaucoma experts and general ophthalmologists and the results of the GPAII EA and TA.

Secondary aims are to evaluate the influence of the stage of the disease on the agreement between clinicians and GPAII, the agreement between glaucoma experts’ and general ophthalmologists’ evaluation, between each pair of assessors and between GPAII EA and GPAII TA, and the sensitivity and specificity of clinicians to detect VF progression, considering GPAII EA and TA as the reference standard.

## 2. Materials & Methods

This was a retrospective study including series of at least 5 visual field tests of glaucoma patients attending the Glaucoma Unit of IRCCS-Fondazione GB Bietti, Rome, Italy, performed in at least 2 years of follow-up. All patients signed the written informed consent form.

The study was approved by the ethical committee of IRCCS-Fondazione GB Bietti (Registro Sperimentazioni N82/19/FB) and agreed with the tenets of the Declaration of Helsinki.

Glaucoma was defined by the presence of the typical glaucomatous visual field defect: a glaucoma hemifield test result outside the normal limits, a mean deviation and pattern standard deviation with *p* < 0.05 probability of being normal, a cluster of at least 3 contiguous points of *p* < 0.05 not contiguous with the blind spot and not crossing the horizontal midline where 1 of the 3 contiguous points with *p* < 0.01 of being normal, on the pattern standard deviation plot.

Only reliable exams were included in the study (false negative < 20%; false positive < 15%; fixation loss < 33%).

Visual field series were not included in the study if patients were under 18 years old, not able to read and sign the written informed consent form, had concomitant ocular disease involving the optic nerve or the macula.

Furthermore, VF series with GPAII report of “possible progression” were excluded because this result suggests repeating the exam to confirm the presence of progression and because the false positive rate of “possible progression” is 34% [8].

Three glaucoma experts (MM, LT and FO) and three general ophthalmologists (MB, LB and LMDG) assessed the progression status of each VF series, using the overview report, thus looking at the grey scale maps, the threshold absolute values maps, the total and pattern deviation probability plots, the MD, PSD and VFI values.

Both groups were not allowed to read the GPAII reports of those VF series.

Glaucoma experts had at least 5 years of experience in a glaucoma center, while general ophthalmologists did not have any experience in a glaucoma center.

The GPAII reports, including both EA and TA, were collected by another glaucoma expert (GR).

The VF series were considered as progressive for both glaucoma experts and general ophthalmologists if two of the three glaucoma experts and two of the three general ophthalmologists stated that there was progression.

The VF series were considered not progressive for both glaucoma experts and general ophthalmologists if two of the three glaucoma experts and two of the three general ophthalmologists respectively stated that there was not progression.

### Statistical Analysis

Normal distribution of the data was investigated with the Shapiro-Wilk test. Since all variables showed a normal distribution, mean ± SD was used for descriptive statistics.

Categorical variables were expressed as percentage and compared using Fisher’s exact test.

The agreement between glaucoma experts’ and general ophthalmologists’ evaluation and both GPAII EA and GPA II TA was reported by the percentage of VF series with discordant opinion, and it was analyzed by using the Cohen’s kappa statistic. Kappa values were classified as fair (0.2 ≤ 0.4), moderate (0.4 ≤ 0.6), good (0.6 ≤ 0.8), and very good (0.8 ≤ 1) [9].

The percentage of VF series with discordant opinion and k statistics was used also to evaluate the agreement between glaucoma experts’ and general ophthalmologists’ evaluation, between each pair of assessors and between GPAII EA and GPAII TA.

Sensitivity and specificity to detect VF progression were calculated for glaucoma experts’ and general ophthalmologists’ evaluation, considering GPAII EA and TA as the reference standard.

## 3. Results

Sixty-six VF series of 44 glaucoma patients were included in the study (one eye of 22 patients and both eyes of other 22 patients).

Each series had a mean of 7.1 ± 1.4 VF with a mean follow-up of 4 ± 1 years. Overall, 468 VF were analyzed.

Forty-three percent of patients were male (19/44), while 57% were female (25/44).

The mean age was 69.9 ± 10.3 years. The mean baseline MD was −4.62 ± 4.26 dB, while the median baseline MD was −3.59 dB.

Seventy-one percent of patients (47/66) had early glaucoma (MD > −6 dB), 21% of patients (14/66) had moderate glaucoma (−12 < MD < −6 dB) and 8% of patients (5/66) had advanced glaucoma (MD < −12 dB). None of the VF with advanced glaucoma was flagged with the alert “Pattern Deviation not shown for severely depressed fields”.

Results of glaucoma experts’ and general ophthalmologists’ evaluation as well as results of the GPAII EA and TA are reported in Table 1.

Sixty-two percent of VF series (41/66) were judged in the same way by glaucoma experts, general ophthalmologists, GPAII EA and GPAII TA.

The clinical judgment of glaucoma experts and general ophthalmologists was discordant with GPAII EA in 27.2% (18/66) and 28.7% (19/66) of the VF series examined, respectively (k = 0.35, 95% CI 0.15–0.56 and k = 0.30, 95% CI 0.09–0.52).

The clinical judgment of glaucoma experts and general ophthalmologists was discordant with GPAII TA in 21.2% (14/66) and 25.7% (17/66) of the VF series examined, respectively (k = 0.51, 95% CI 0.31–0.72 and k = 0.41, 95% CI 0.18–0.62).

The percentage of VF series with discordant opinion and the k statistics between glaucoma experts’ and general ophthalmologists’ evaluation and GPA II EA and TA considering VF series with baseline MD greater or less than baseline median MD (−3.59 dB) is reported in Table 2.

The clinical judgment of glaucoma experts and general ophthalmologists was discordant in 16.7% (11/66) of the VF series examined (k = 0.64, 95% CI 0.46–0.84), while results of GPAII EA and GPAII TA were discordant in 9% (6/66) of the VF series examined (k = 0.69, 95% CI 0.47–0.92).

The percentage of VF with discordant opinion and the k statistics between each pair of assessors (among glaucoma experts and general ophthalmologists) is reported in Table 3.

Considering the GPAII EA as the reference standard, glaucoma experts showed a sensitivity of 90% (9/10 VF series) and a specificity of 69.6% (39/56 VF series), while general ophthalmologists showed a sensitivity of 80% (8/10 VF series) and a specificity of 69.6% (39/56 VF series) to detect VF progression.

Considering GPAII TA as the reference standard, glaucoma experts showed a sensitivity of 92.8% (13/14 VF series) and a specificity of 75% (39/52 VF series) while general ophthalmologists showed a sensitivity of 78.5% (11/14 VF series) and a specificity of 73% (38/52 VF series) to detect VF progression.

## 4. Discussion

The evaluation of VF progression is critical in the management of patients’ follow-up. When progression is evident, clinicians can increase medical therapy or schedule surgery, thus affecting patients’ quality of life. However, to date, there is not a reference method for determining whether there is VF progression or not.

In clinical practice, one commonly utilized approach is the subjective assessment of serial VF printouts, regardless of whether the clinician is a glaucoma expert or a general ophthalmologist. The assessment of serial VF printouts includes the evaluation of all the data available in the printout of the exam: the grey scale, the threshold absolute values, the total and pattern deviation plots, the perimetric indices.

Clinical judgment is difficult due to several variable factors: the short and long-term fluctuation, the influence of cataract or corneal opacities on general sensitivity threshold, the influence of macula diseases on the fovea sensitivity, the learning effect and, mostly, the lack of standardized criteria to define progression [6,7].

Furthermore, it is time consuming especially when the series consists of many VF.

To overcome these limits different types of statistical analysis have been introduced [10,11,12,13]. The GPAII is a statistical software available in the Humphrey Field Analyzer (Carl Zeiss, Dublin), the most popular perimeter used to follow the functional damage of glaucoma patients.

As mentioned before, the GPAII provides both an EA and a TA.

The EA has been developed based on the methods and results of the EMGT Trial. The TA is based on the yearly rate of change of the VFI. It employs regression analysis to assess deterioration rate [4,5].

In this study, we assessed the agreement between a team of three glaucoma experts and a team of three general ophthalmologists with both GPAII EA and TA to detect glaucoma progression.

Both teams of clinicians found progression in a statistically higher percentage of VF series than the two GPAII analyses (39% and 38% for glaucoma experts and general ophthalmologists, 15% and 21% for GPAII EA and TA).

The agreement between both teams of clinicians and GPAII EA was fair, while the agreement between both teams of clinicians and TA was moderate. This difference may be related to the possibility that clinicians had to read the VFI values on the overview report, thus estimating the trend of VFI over time, as reported in the GPAII TA.

Similar results were described by Tanna et al., who found fair agreement (k = 0.52) between expert consensus and glaucoma progression event analysis (GPA) [14]. Expert consensus was also more likely to classify a series of fields as showing progression than was GPA. Nevertheless, Anton et al. found very good agreement (k = 0.82) between glaucoma experts and GPAII EA [15]. However, in the study by Tanna et al., like in our study, glaucoma specialists were not given any guidelines for evaluating the VF series, while in the study by Anton et al., clinicians were instructed to use three methods to assess progression, two of which were based on event analysis.

Since the distribution of the stage of the disease according to the Hodapp–Parrish–Anderson criteria was not homogeneous, we divided the VF series respect to the baseline median MD values (−3.59 dB), to investigate the influence of the stage of the disease on the agreement between clinicians and GPAII.

The agreement between glaucoma experts and GPAII EA was moderate when evaluating VF series with early defect (MD > −3.59 dB), while it was fair when evaluating VF series with worse defect (MD < −3.59 dB).

Similarly, Tanna et al. found smaller agreement between expert consensus and GPA in the subsets of field with MD < −10 dB [14].

In our study, VF series with MD < −3.59 dB included VF with a mean range of defect from −3.60 dB to −15.69 dB: glaucoma experts found progression in a higher percentage of VF series compared to GPAII EA (12/33, 36% vs. 3/33, 9%).

Otherwise, the agreement between glaucoma experts and GPAII TA was moderate when evaluating both VF series across the median MD value (−3.59 dB).

The agreement between general ophthalmologists and both GPAII EA and TA was moderate when evaluating VF series with MD > −3.59 dB, while it was fair when evaluating VF series with MD < −3.59 dB. Again, when evaluating VF series with MD < −3.59 dB, clinicians found higher percentage of progression (42%,14/33, of VF series) compared to the GPAII EA (9%, 3/33, of VF series) and to the GPAII TA (18%, 6/33, of VF series).

Overall, results suggested that glaucoma experts showed better agreement with GPAII when evaluating VF series with baseline MD <−3.59 dB, compared to general ophthalmologists.

It is important to point out that when looking at the overview report, clinicians could consider several factors to determine progression which are not considered by the GPAII, such as the location of deteriorated points in critical areas for vision.

Furthermore, the limits of both GPAII EA and TA should be considered.

The GPAII EA is not able to detect progression in cases of advanced glaucoma and is not sensitive in detecting focal changes occurring in one single point that worsens more than 10 dB [16,17].

The GPAII TA is affected by a ceiling effect of the VFI: for VF with MD value around −5 dB the VFI can be close to its maximum value of 100% and so it is not able to detect early visual field loss [18]. On the other side, in VF with MD value over −20 dB the VFI increases its variability, thus reducing its reliability [19,20].

These limits may justify why in our study GPAII was more conservative than clinicians when analyzing VF with moderate and advanced defects and suggest that the software cannot replace the clinical examination of each VF.

Interestingly, the agreement between glaucoma experts and general ophthalmologists was good (k = 0.64, 95% CI 0.46–0.84). Considering the agreement between each pair of assessors, we found k values from 0.65 to 0.78 (good) among glaucoma experts and k values from 0.23 (fair) to 0.9 (very good) among general ophthalmologists. Probably, glaucoma experts’ evaluation was more consistent compared to that of general ophthalmologist due to the similar training and background of the three assessors.

Similarly, in the study by Iester et al., the inter-observer agreement among nine glaucoma specialists, when using an overview report for detecting glaucoma progression, was good with k values ranging from 0.46 to 0.70 [21]. On the other side, in the study by Viswanathan et al., the agreement between five glaucoma specialists in detecting VF progression was fair (k = 0.32) when using Humphrey printouts [22]. However, in our study and in the study by Iester et al., the mean number of VF in the series evaluated by clinicians was respectively 7.1 ± 1.4 and 7.9 ± 3.4, while in the study by Viswanathan et al., clinicians were presented VF series with at least 16 exams. Thus, the difference in series length appears to have influenced the level of agreement between clinicians using the overview report of the Humphrey printouts. In fact, longer periods of VF follow-up may make the decision about progression status more complex and difficult.

The two GPAII analysis are based on different algorithm, nevertheless, the agreement between them in our study was good (k = 0.69, 95% CI 0.47–0.92). Rao et al., instead, reported a moderate agreement between the two analysis [23]. However, in our study, we defined progression on TA as any VFI slope with a *p* value < 0.05, while Rao et al. also considered a slope magnitude of −1% per year as clinically significant.

In our study, when considering GPAII EA as the reference standard, glaucoma experts showed higher sensitivity than general ophthalmologists (90% vs. 80%) to detect VF progression, as well as when considering GPAII TA as reference standard (92.8% vs. 75%).

This study has two main limitations. First, the 95% CI for the kappa values are quite wide, so the estimation of the agreement may be a little imprecise. Therefore, we also reported the percentage of VF series with discordant opinion. Second, the VF series were not homogenous regarding the stage of the disease (few VF series with advanced defect). However, the evaluation of the influence of the stage of the disease on the agreement between clinicians and GPAII was supposed to be a secondary aim of the study.

In conclusion, the software appears to be more conservative than clinical judgment in assessing the presence of VF progression, especially when evaluating VF series with more advanced defects. Several factors affect the agreement between the clinical evaluation of glaucoma experts, general ophthalmologists, and GPAII EA and TA in detecting VF progression. Since all the methods have strengths and weaknesses, it would be interesting to explore if combining clinical judgment and GPAII would be more accurate in evaluating VF progression, rather than considering only one method.

Furthermore, glaucoma experts showed better agreement with GPAII when evaluating VF series with moderate and advanced glaucoma and a better ability in detecting VF progression, with respect to general ophthalmologists.

## Figures and Tables

**Table 1:** Percentage of VF series with or without progression according to both glaucoma experts’ and general ophthalmologists’ evaluation and both GPAII event and trend analysis.

	Progression	Not Progression
Glaucoma experts	39%	61%
	(26/66)	(41/66)
General ophthalmologists	38%	62%
	(25/66)	(41/66)
Guided Progression Analysis II- Event Analysis (GPAII EA)	15%	85%
	(10/66) *^,^ ***	(56/66)
Guided Progression Analysis II-Trend Analysis (GPAII TA)	21%	79%
	(14/66) **^,^ ****	(52/66)

Fisher’s exact test result is statistically significant (*p* < 0.05) between glaucoma experts and both GPAII EA (*) and GPAII TA (**) and between general ophthalmologists and both GPAII EA (***) and GPAII TA (****).

**Table 2:** Percentage of VF series with discordant opinion and k statistics with 95%CI between GPAII EA and TA and glaucoma experts’ and general ophthalmologists’ evaluation considering VF series with baseline MD greater or less than baseline median MD (−3.59 dB).

	MD > −3.59 dB (Range 0.8/−3.58 dB)		MD < −3.59 dB (Range −3.60/−15.69 dB)	
	Guided Progression Analysis II-Event Analysis (GPAII EA)	Guided Progression Analysis II-Trend Analysis (GPAII TA)	GPAII EA	GPAII TA
Glaucoma experts	27.2% (9/33)	24.2% (8/33)	27.2% (9/33)	18.1% (6/33)
	0.40	0.47	0.29	0.56
	(0.11–0.70)	(0.18–0.76)	(0.01–0.58)	(0.27–0.84)
General ophthalmologists	18.1% (6/33)	21.2% (7/33)	39.3% (13/33)	30.3% (10/33)
	0.55	0.48	0.10	0.32
	(0.24–0.85)	(0.16–0.80)	(−0.13–0.33)	(0.04–0.61)

**Table 3:** Percentage of VF series with discordant opinion and k statistics with 95%CI between each pair of assessors among glaucoma experts and general ophthalmologists.

	Assessors		Assessors		Assessors	
Glaucoma	#1 vs. #2	16.6%	#2 vs. #3	15.1%	#1 vs. #3	10.6%
experts		(11/66)		(10/66)		(7/66)
		0.65		0.69		0.78
		(0.45–0.84)		(0.51–0.87)		(0.62–0.93)
General	#1 vs. #2	4.5%	#2 vs. #3	39.3%	#1 vs. #3	40.9%
ophthalmologists		(3/66)		(26/66)		(27/66)
		0.9		0.29		0.23
		(0.8–1)		(0.11–0.46)		(0.04–0.43)

## Data Availability

The original data used and analyzed to support the findings of this study are included in the Supplementary Materials, further inquiries can be directed to the corresponding author.

## Supplementary Materials

The following supporting information can be downloaded at: https://www.mdpi.com/article/10.3390/jcm11195508/s1, Table S1: Dataset of patients enrolled in the study.
